# Regional variation in ambulatory care-sensitive hospitalizations for people with type 2 diabetes in Germany: insights from a claims data analysis using the PopGrouper

**DOI:** 10.1007/s43999-025-00082-0

**Published:** 2025-12-17

**Authors:** Chrissa Tsatsaronis, Anika Kreutzberg, Maria Klemt, Karen Kinder, Reinhard Busse, Wilm Quentin

**Affiliations:** 1 Department of Health Care Management, Technische Universität Berlin, Berlin, Germany; 2 Department for Planetary and Public Health, Universität Bayreuth, Bayreuth, Germany

**Keywords:** Diabetes, Ambulatory care sensitive condition, Population classification, Regional variation, Risk adjustment, Germany

## Abstract

**Background:**

Diabetes-related hospitalizations and amputations serve as indicators of healthcare system performance, revealing regional disparities in diabetes management. This study examines regional variations in diabetes-related and all-cause outcomes among people with type 2 diabetes across Germany, adjusting for morbidity using the novel PopGrouper classification system. The paper aims to test the applicability of the PopGrouper for analyses of regional variations in health care.

**Methods:**

Based on claims data from the BARMER sickness fund, six outcomes (hospitalizations, emergency hospitalizations, amputations, hospital care costs, inpatient days, and mortality) were analyzed. For each outcome, the ratio of observed to expected outcomes was calculated based on each region’s PopGroup composition and compared to results based on age-sex composition. Finally, the analysis identified top- and bottom-performing regions based on PopGroup-adjusted performance and explored patterns of resource utilization in these regions.

**Results:**

The findings reveal the greatest regional variation for amputations and emergency hospitalizations, followed by hospitalizations, for both, age-sex-standardized and PopGroup-standardized ratios. The observed variation was slightly lower with PopGroup standardization than with age-sex standardization, except in the case of mortality. The top-performing regions had lower inpatient resource utilization, but higher outpatient resource utilization and participation in disease management programs in the year prior to the performance outcome measurement.

**Conclusion:**

Regional variations in hospitalizations and related outcomes for type 2 diabetes were observed. The PopGrouper enables straightforward risk-adjustment through indirect standardization and can make a significant contribution to controlling for morbidity-related care needs in regional comparative analyses.

**Supplementary Information:**

The online version contains supplementary material available at 10.1007/s43999-025-00082-0.

## Introduction

Diabetes is one of the most common chronic illnesses, affecting more than 500 million people worldwide. The global prevalence of diabetes is projected to increase, which poses a growing challenge to population health [1]. In Germany, more than 9.4 million people (approximately 11% of the population) are diagnosed with diabetes, with more than 9.1 million affected by type 2 diabetes [2]. Type 1 and type 2 diabetes are both characterized by elevated blood glucose levels, but differ in their underlying mechanisms and treatment approaches: Type 1 diabetes is an autoimmune condition and cannot be prevented, whereas type 2 diabetes is often preventable [3].

Diabetes (types 1 and 2) can be effectively managed in outpatient care settings through routine monitoring, patient education, and timely intervention [4]. When adequately controlled, many of the condition’s complications — including hospitalizations and diabetes-related amputations — can be avoided [5–7]. As such, diabetes is classified as an ambulatory care-sensitive condition (ACSC), meaning that high-quality primary care can often avert disease progression and the need for hospital admission. Rates of diabetes-related hospitalizations and amputations, are widely used as indicators to assess and compare health system performance, including in international benchmarks, such as OECD reports [7, 8]. Elevated rates of hospitalizations and amputations may indicate problems with accessibility or quality of primary health care [9, 10], and can thus help identify regional disparities in diabetes care delivery.

Previous research has documented regional variations in diabetes care and related outcomes both internationally [4, 8, 11–14] and in Germany [15–19]. These variations include differences in diabetes-related hospital admission rates [4, 8, 11, 15, 16, 19], emergency hospital admissions [12], and amputation rates [8, 16–18], as well as all-cause hospitalizations [13] and all-cause mortality [14]. However, such outcomes are influenced not only by health system factors, but also by the underlying characteristics of the patient population. The population of individuals with diabetes is highly heterogeneous, with many patients experiencing multiple comorbid conditions — some related to diabetes and others not [20]. To enable meaningful assessment of regional differences, it is essential to account for this heterogeneity through appropriate risk adjustment. While many studies adjust for age and sex [4, 8, 17–19], fewer account for comorbidity measures, despite their relevance for capturing the complexity of diabetes patients [11, 21].

In this paper, we explore regional variation in health care outcomes among people with type 2 diabetes in Germany, using the PopGrouper to adjust for morbidity. The PopGrouper is a novel population-based classification system which classifies persons into mutually exclusive groups that consider multimorbidity and exhibit a high degree of morbidity differentiation. As a result, individuals in the same group have similar medical needs and costs, thus enabling regional comparisons for groups of patients with similar needs [22]. Other such population-based groupers exist internationally and are used, for instance, to describe the burden of disease of the population, analyze trends over time, and compare populations across different regions [23–25].

This paper aims to (1) quantify regional variation in diabetes-related efficiency and quality outcomes among people with type 2 diabetes across Germany and to assess how this variation changes when adjusting for morbidity using the PopGrouper; (2) identify regions that perform above or below average based on PopGroup-adjusted performance; and (3) explore patterns of care and resource utilization in top- and bottom-performing regions. The overall aim is to test the PopGrouper’s applicability and highlight its possible advantages for the assessment of regional health care variation.

## Methods

### Data and measures

#### Study population

The analysis is based on pseudonymized claims data from the BARMER sickness fund from the years 2022 and 2023. BARMER covers approximately 10% of the German population (8.8 million in 2022), making it one of the largest German sickness funds, and has presence in all German federal states. The data was accessed via the BARMER Scientific Data Warehouse, which comprises data on demographics, inpatient and outpatient services, rehabilitation services, prescription drugs, remedies, and medical aids for all individuals insured by BARMER. Analysis was performed using SAS Enterprise Guide 8.4.

The study population included individuals aged 18 or older who were continuously insured by BARMER in 2022, had a documented diagnosis of type 2 or unspecified diabetes that year, and were either continuously insured or deceased in 2023. The decision to focus on type 2 diabetes was made to ensure a more specific study population, and because type 2 diabetes accounts for the vast majority of diabetes cases in Germany. The diagnosis selection criteria were adopted from the German morbidity-based risk adjustment scheme used to compensate for differences in risk structures among sickness funds [26]. The detailed list of diagnosis selection criteria is included in Supplementary Material 1. Individuals were excluded if they additionally had a documented type 1 diabetes diagnosis (E10) or had missing or invalid ID, age or postal code information.

#### Outcome parameters

Six efficiency and quality outcomes were defined and measured per person in the year 2023: (1) Hospitalization with a principal diabetes diagnosis (dummy variable), (2) emergency hospitalization with a principal diabetes diagnosis (dummy variable), (3) minor or major amputation (German procedure codes (OPS): 5-864 or 5-865 [27]) (dummy variable), (4) total cost of hospital care (for all admissions, in Euros), (5) total number of inpatient treatment days, and (6) all-cause mortality (dummy variable).

#### Risk adjustment with PopGrouper

Each person in the study population was assigned to their corresponding PopGroup, applying the PopGrouper version 1.0 using all diagnoses and characteristics from the year 2022. This version includes a total of 776 PopGroups. Every PopGroup comes with a composite severity score which reflects the relative complexity and severity of that group. The severity score is calculated using the standardized mortality ratio, average healthcare utilization (hospital days and outpatient cases), and average costs associated with that group. In the PopGrouper 1.0, the severity score ranges from -0.4 to 13.1, with a higher score indicating a higher severity [22].

#### Regions

Each person was assigned to a region based on their place of residence. The 96 German spatial planning regions (Raumordnungsregionen) represented the regional unit of analysis [28]. Each region was classified into one of three levels of urbanization: urban region, region with intermediate urbanization, or rural region. This classification is based on the proportion of the population residing in large and medium-sized cities, the presence and size of large cities, overall population density, and population density excluding large cities [29]. Additionally, each region was assigned a level of deprivation according to the German Index of Socioeconomic Deprivation (GISD). The currently available version is based on data from 2019. The GISD serves as a measure of relative regional socioeconomic deprivation and can range between 0 (lowest deprivation) and 1 (highest deprivation). Regions were then grouped into five categories based on GISD quintiles [30].

### Analysis

#### Descriptive analysis

In a first step, the study population was described in its demographic and socioeconomic composition. Maps were used to depict the regional distribution of the diabetes population and the regional distribution of the PopGroup composite severity score. Five groups were defined for each map using quintiles.

#### PopGroup-standardized observed to expected ratios

To quantify regional variation in outcomes, the methods proposed and detailed by Kreutzberg et al. were applied [22]. PopGroups are mutually exclusive and can therefore be used to calculate observed to expected (O/E) ratios. The O/E ratio represents the ratio of the observed value (e.g., number of events or average for continuous variables) for a given outcome variable in a region to the expected value for this outcome based on a reference population (in this case the national average), usually adjusted for risk. It serves as a measure of relative regional performance compared to the national average (O/E = 1). An O/E ratio of 1.2 indicates that the observed outcome is 20% higher than expected for the region’s patient population, while an O/E ratio of 0.8 indicates an outcome 20% lower than expected [31].

First, age and sex groups (with age groups defined in 5-year intervals) were used to calculate age-sex-standardized O/E ratios, following the approach commonly used for standardized mortality ratios [32]. Subsequently, using the same standardization approach, PopGroup-standardized outcome ratios were calculated to account for the different PopGroup distribution in each region. Assuming that a patient’s treatment outcomes are associated with their level of multimorbidity and health care utilization – which is reflected in the PopGroup – the PopGroup-standardized outcome ratio can be interpreted as a morbidity-adjusted comparison of regional performance.

#### Graphical analysis

To visualize the level of regional variation in outcomes, distribution diagrams were plotted in which the 96 regions were sorted by their age-sex-standardized O/E ratio. The reference line at 1 represents the national average. Regions plotted below the reference line represent regions with better outcome performance than the national average and vice versa. The distribution diagrams help visualize the level of regional variation and detect patterns in regional performance across different outcomes. Additionally, the distribution of regions based on their relative performance can be visually compared across the different O/E ratio measures (age-sex-standardized and PopGroup-standardized) to show how the relative regional performance changes after accounting for demographics and multimorbidity. The PopGroup-standardized results are also presented in maps to illustrate the spatial patterns of the outcomes. For each map, five groups were defined based on quintiles.

#### Comparison of regional utilization patterns

Finally, utilization patterns from the year preceding the outcome performance were analyzed in the top- and bottom-performing regions to descriptively explore the relationship between healthcare utilization in one year and performance in the next, and to look for possible patterns between the higher- and lower-performing regions. To do this, a set of utilization variables measured in 2022 (prior to the performance outcomes measured in 2023) were analyzed. These variables included the number of hospitalizations, inpatient costs, number of outpatient cases, outpatient costs, number of inpatient and outpatient emergencies, and participation in a disease management program for diabetes. The regions were ranked based on their mean PopGroup-standardized O/E ratio for selected quality and efficiency outcomes, to identify top- and bottom-performing regions that fell 20% above or below the national average. The average utilization in the top-performing regions was then compared to the national average and to the bottom-performing regions.

## Results

### Descriptive results

#### Study population

In total, 745,741 BARMER insured individuals with diabetes were included in the study population. Table 1 shows the demographic characteristics of the study population. The population was 55% female and mostly aged above 60 (84%). Nearly half of the population resided in an urban region (45%). Approximately one quarter (26%) resided in a region of medium deprivation according to the GISD, with the rest fairly evenly distributed across the other four deprivation categories [30].

**Table 1:** Description of study population

	*N*	%
**Total study population**	745,741	100.0
**Sex**		
Male	337,825	45.3
Female	407,916	54.7
**Age group**		
Age 18–59	119,031	16.0
Age 60–69	178,761	24.0
Age 70–79	224,770	30.1
Age >= 80	223,179	29.9
Level of urbanization*		
Urban region	331,950	44.5
Intermediate urbanization	215,572	28.9
Rural region	198,219	26.6
Level of deprivation**		
Lowest deprivation	128,120	17.2
Second lowest deprivation	139,688	18.7
Medium deprivation	194,829	26.1
Second highest deprivation	148,174	19.9
Highest deprivation	134,930	18.1

Note: * 2022, ** 2019. Level of urbanization based on Federal Institute for Research on Building, Urban Affairs and Spatial Development [29]. Level of deprivation based on the German Index of Socioeconomic Deprivation (GISD) in 2019 [30]. For better presentation, four approximately equal-sized age groups were defined for this table, instead of using 5-year intervals

A total of 663 PopGroups were present in the population. The 10 most frequent PopGroups are listed in Table 2. Combined, the two most frequent PopGroups (“*Diabetes mellitus with disease symptoms affecting at least one organ system + at most 14 Major Disease Groups and level of care dependency ≤ 2*” and “*Diabetes mellitus without complications + at most 7 Major Disease Groups and aged 30 years or older*”) accounted for a quarter of the study population. The remaining three quarters were distributed among the remaining 661 PopGroups, the majority of which are not defined by the presence of a diabetes diagnosis. Instead, they are characterized by other dominant diseases, such as lung disease and heart disease in the case of the fourth and fifth PopGroups.

**Table 2:** Top 10 PopGroups in the study population

Rank	PopGroup	PopGroup description	Severity	*N*	%
1	**P06045BB**	Diabetes mellitus with disease symptoms affecting at least one organ system + at most 14 MDGs and level of care dependency ≤ 2	-0.07	101,814	13.7
2	**P07053BB**	Diabetes mellitus without complications + at most 7 MDGs and aged 30 years or older	-0.23	92,034	12.3
3	**P07021ZZ**	Diabetes mellitus without complications & Osteoarthritis or osteochondrosis	-0.12	21,768	2.9
4	**P06063BB**	Diseases of the lungs (MDG) & Diseases of the heart (MDG) + at most 12 MDGs and level of care dependency ≤ 2	-0.15	17,258	2.3
5	**P07040BB**	Coronary sclerosis or other chronic ischemic heart disease + at most 8 MDGs and level of care dependency ≤ 1	-0.21	13,696	1.8
6	**P06034BB**	Neurological diseases (MDG) & Chronic pain + at most 15 MDGs without long-term insulin treatment	0.02	11,933	1.6
7	**P03061BB**	Acute respiratory failure + at most 5 (very) severe MDGs with mechanical ventilation ≤ 499 h	1.02	9,944	1.3
8	**P06023BB**	Severe depression + at most 14 MDGs and age < 18 years	0.16	9,377	1.3
9	**P05106BB**	Other (very) severe condition(s) + at most 13 MDGs without long-term insulin treatment	-0.20	8,421	1.1
10	**P07053AZ**	Diabetes mellitus without complications + more than 7 MDGs	-0.16	8,256	1.1

Note: Ranking by the number of individuals in each PopGroup, in descending order. The severity is measured using the PopGroup composite severity score, whereby a higher score indicates a higher severity. MDG: Major Disease Group. The MDGs are defined as part of the PopGrouper and classify the consolidated disease groups (CDGs) into mutually exclusive diagnosis areas. The level of care dependency classifies patients based on their dependency on long-term care and ranges from 0 (no care dependency) to 5 (highest level of care dependency) [33]. The first 3 digits of the PopGroup denote the overarching Macro PopGroup (MPG): P03: Severe high-cost cases, P05: At least one (very) severe condition, P06: At least one moderate condition, P07: At least one mild condition [33]

#### Outcome parameters

In 2023, 1.1% (*n* = 8,044) of the study population was hospitalized due to diabetes, 0.7% (*n* = 5,355) had an emergency admission for diabetes, 0.4% (*n* = 2,904) had a major or minor amputation, and 5.1% died (*n* = 38,119). The mean number of all-cause hospital treatment days was 4 days (range from 0 to 364 days), and average total inpatient costs were 3,324 Euros per person (range from 0 to 1,685,663 Euros).

All outcomes are measured for the entire study population, which includes individuals who died in 2023 and thus had a shorter observation period. Table 3 compares outcome parameters between individuals who survived and those who died in 2023. Among the deceased, it further distinguishes between those who died in the first and second halves of the year, to assess whether those observed for less than half of the year might show lower utilization and costs solely due to the shorter observation period. However, individuals who died in 2023, including those who died in the first half of the year, had higher healthcare utilization and costs than those who survived.

**Table 3:** Comparison of individuals who survived and died in 2023

	Study population		Survived in 2023		Died in 2023		Died after ≤ 183 days		Died after > 183 days	
	*N*	%	*N*	%	*N*	%	*N*	%	*N*	%
**Number of persons**	745,741	100.0	707,622	94.9	38,119	5.1	19,267	2.6	18,852	2.5
**Outcomes**	**N**	**%**	**N**	**%**	**N**	**%**				
Hospitalization	8,044	1.1	6,769	1.0	1,275	3.3	482	2.5	793	4.2
Emergency	5,355	0.7	4,332	0.6	1,023	2.7	404	2.1	619	3.3
Amputation	2,904	0.4	2,262	0.3	624	1.7	272	1.4	370	2.0
	**Mean**	**SD**	**Mean**	**SD**	**Mean**	**SD**				
Total costs (Euros)	6,804	13,222	6,170	11,864	18,565	25,719	13,761	21,825	23,474	28,338
Inpatient costs (Euros)	3,324	10,982	2,732	9,474	14,320	23,785	11,331	21,094	17,374	25,899
Inpatient treatment days	4	12	3	11	17	22	12	17	21	26

Note: SD: Standard deviation

#### Regional distribution of case rates and PopGroup severity

**Fig. 1:**
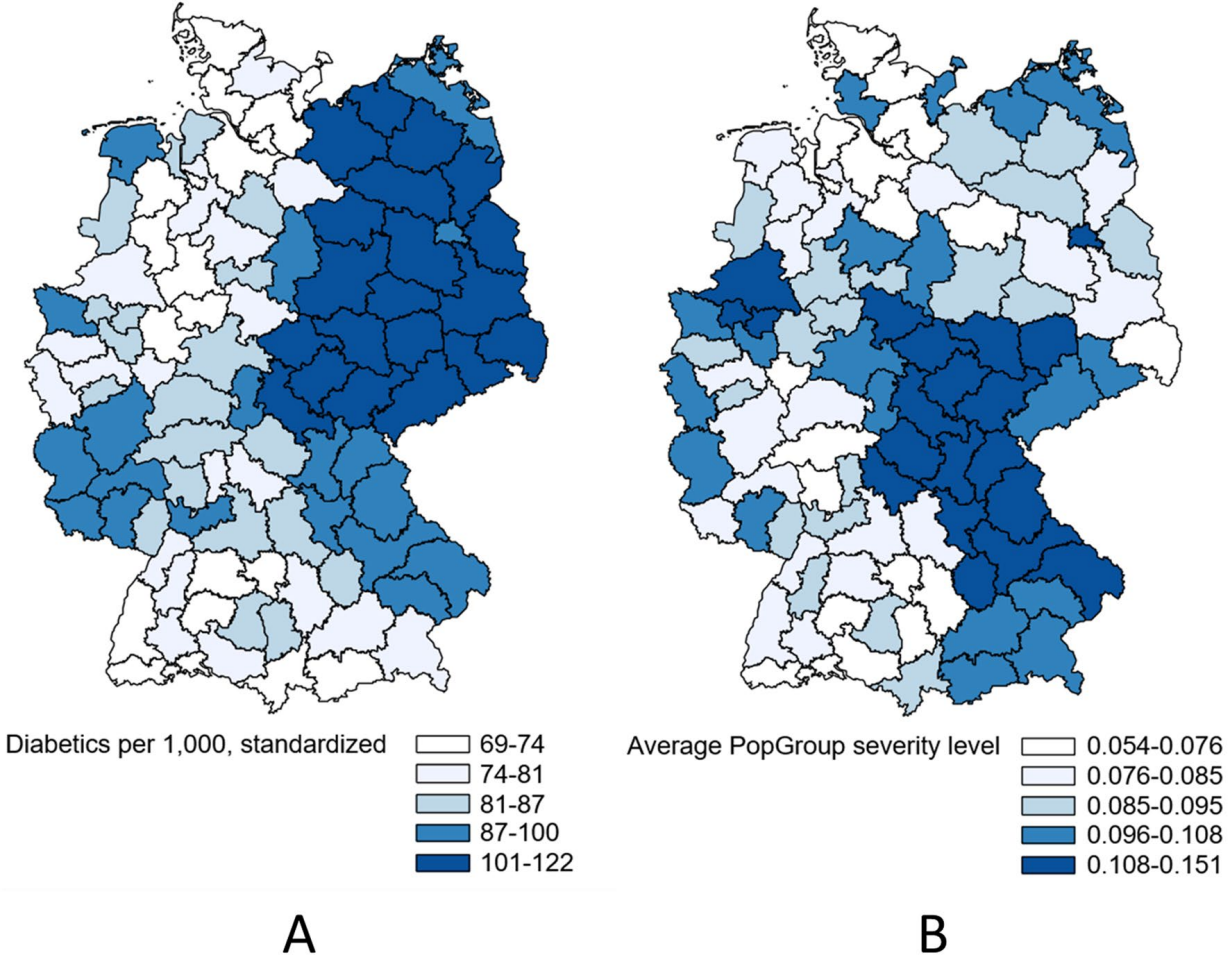
Diabetes case rates per 1,000 BARMER insured individuals per region, age-sex-standardized (panel **A**) and average PopGroup severity level of diabetes patients per region (panel **B**). Note: Based on BARMER insurance data from 2022

On average, there were 7,768 individuals with diabetes in each region, ranging from 1,336 to 37,989. Figure 1A visualizes the age-sex-standardized regional distribution of persons with diabetes per 1,000 BARMER insured individuals in 2022, with darker regions indicating higher case rates. The case rate ranged from 59 to 172 persons with diabetes per 1,000 insured, with higher case rates observed in eastern Germany. Figure 1B shows the average PopGroup composite severity score of diabetes patients per region. The severity score ranged between 0.05 and 0.15 (mean = 0.09). The figure shows that regions in the central and southeastern part of the country have a PopGroup composition with higher severity. Thuringia, southern Saxony-Anhalt, and north-eastern Saxony had both high rates of patients with diabetes and higher than average severity.

### Regional variation in observed to expected outcome

**Fig. 2:**
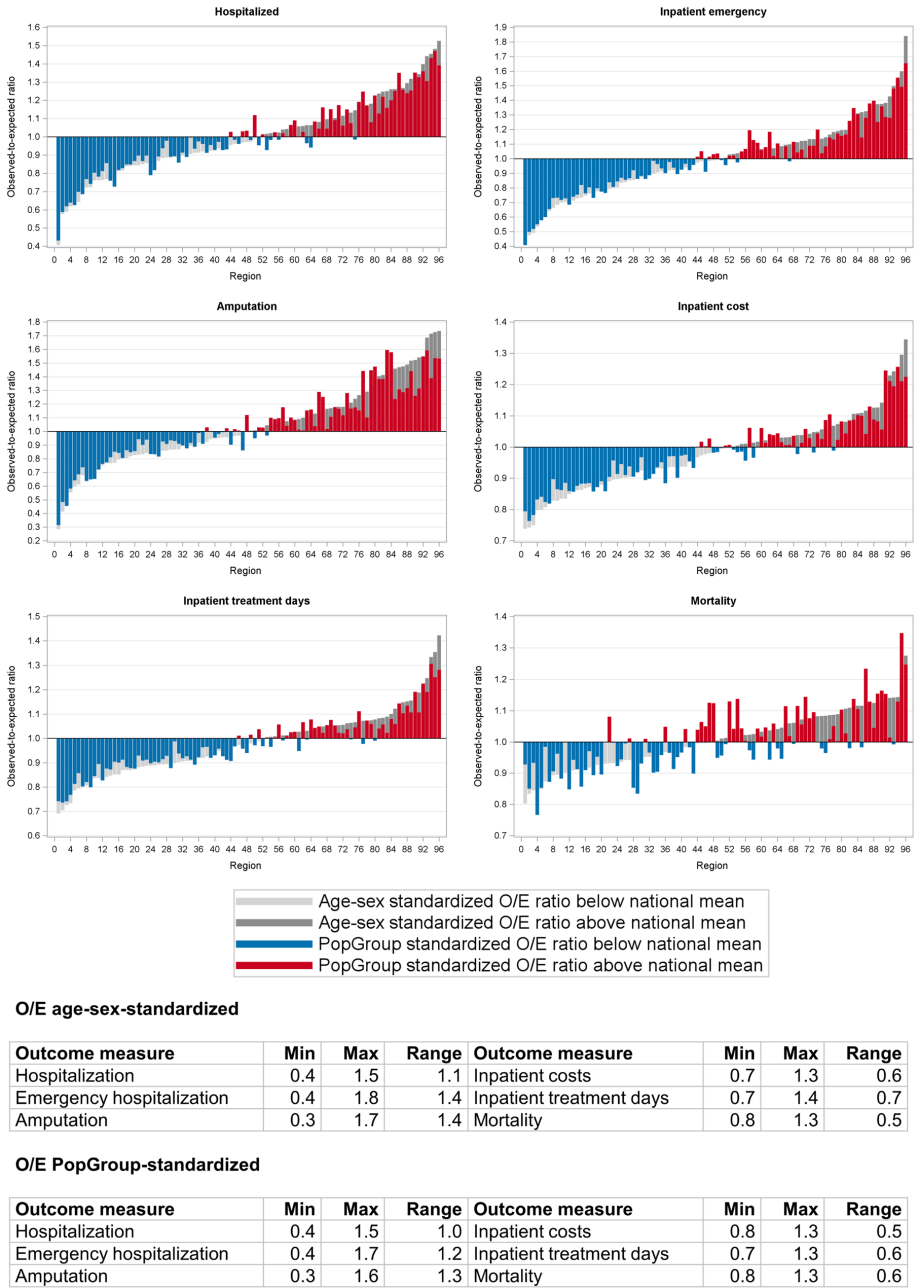
Age-sex-standardized and PopGroup-standardized O/E ratios for each outcome. Note: Regions are ranked by age-sex-standardized O/E ratio. The reference line at 1.0 corresponds to the national mean. O/E: observed to expected ratio

Figure 2 shows the distribution of the age-sex-standardized and PopGroup-standardized outcome ratios for the 96 regions and each outcome. Regions with a PopGroup-standardized outcome ratio above the national average (O/E > 1) are shown in red, while regions below the national average (O/E < 1) are shown in blue. The hidden light and dark grey bars represent the age-sex-standardized outcome ratio for each region. In each graph, the regions are ranked by the age-sex-standardized outcome ratios. This means that regions with a blue bar but on the right side of the graph have moved from an above average to a below average value after PopGroup standardization, and vice versa. The data tables in the figure show the minimum, maximum and range for both the age-sex-standardized and PopGroup-standardized outcome ratios. The range represents the difference in outcome ratios between the best and worst performing regions.

Amputation rates and emergency hospital admissions display the greatest variation between regions, both in the age-sex- and PopGroup-standardized outcome ratios (amputation: 0.3–1.7 and 0.3–1.6; emergency admissions: 0.4–1.8 and 0.4–1.7), followed by hospital admissions (0.4–1.5). The observed variation was slightly lower with PopGroup standardization than with age-sex standardization, except in the case of mortality. The graphs reveal regions that shifted from above average to below average values, and vice versa, following PopGroup standardization. The greatest shifts are observed for mortality (Fig. 2).

**Fig. 3:**
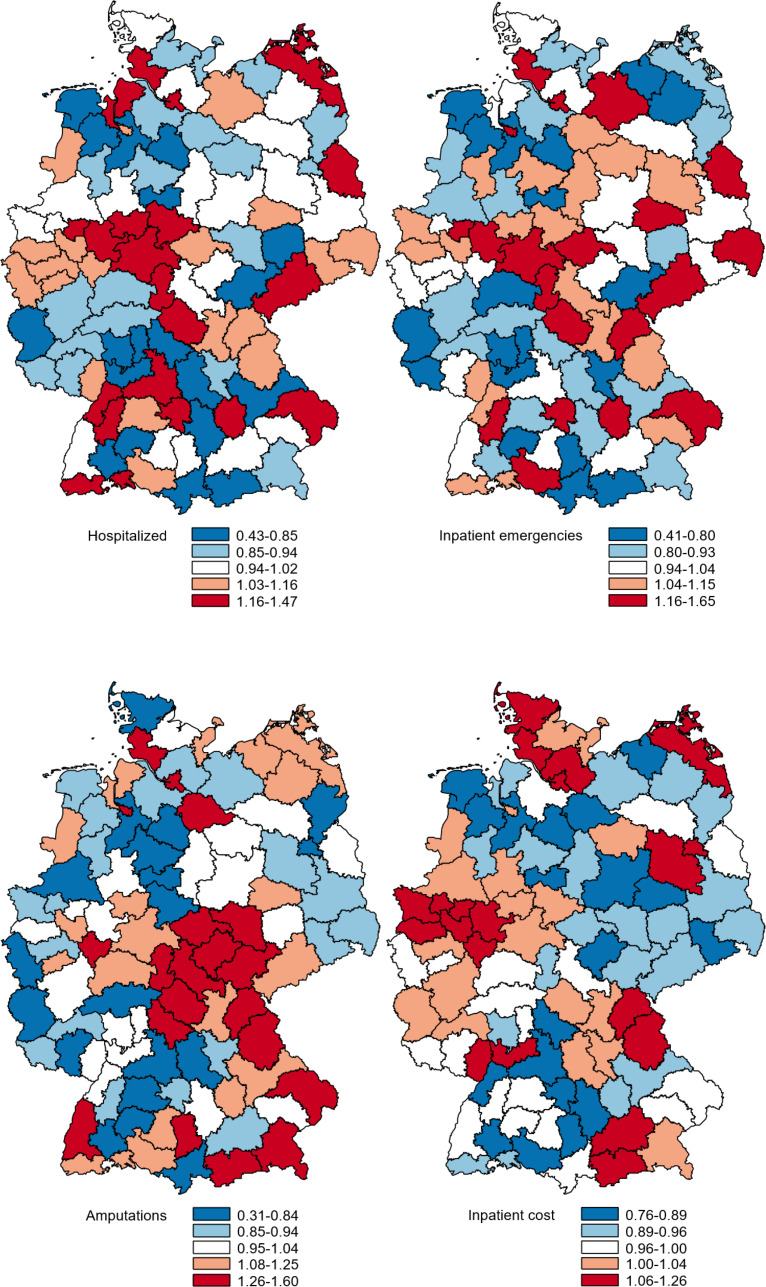

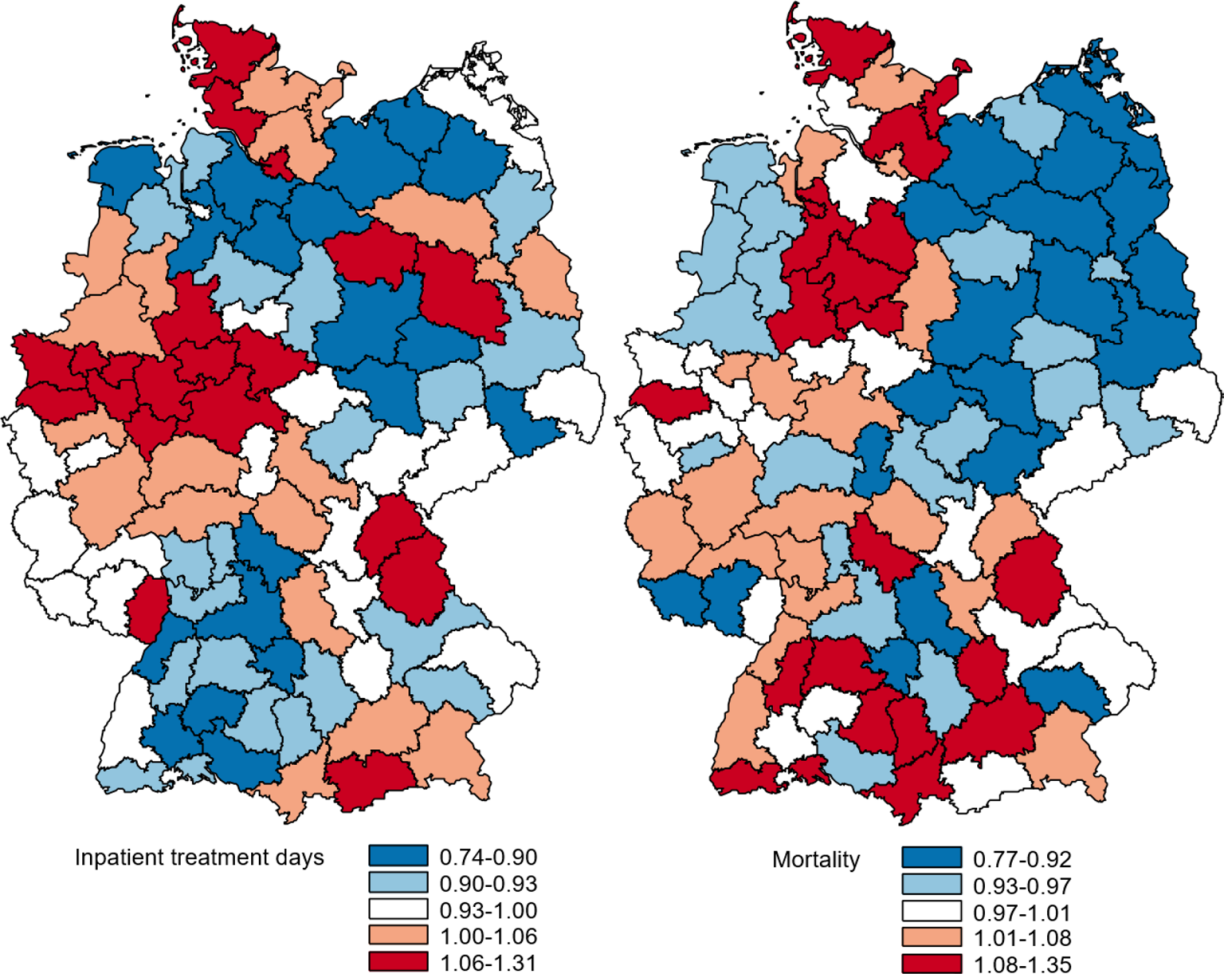
PopGroup-standardized O/E ratios by region

Figure 3 shows the PopGroup-standardized outcome ratios in maps to illustrate the spatial patterns, with red regions indicating outcome values above average and blue regions indicating outcome values below average. The maps indicate that the regions performing higher or lower vary slightly across the different outcomes. Furthermore, a comparison of Fig. 3 with Fig. 1 reveals no apparent association between outcomes and either case rate or severity level.

### Utilization patterns of top- and bottom-performing regions

Based on selected PopGroup-standardized quality and efficiency outcomes, the most and least efficient regions (TopEff, BottomEff) and the regions with the highest and lowest quality of care (TopQual, BottomQual) were identified to compare health care utilization patterns between them in the year preceding the outcome performance. Regions were identified that fall above or below a pre-specified O/E ratio of 1.2 or 0.8 (20% above or below average). Efficiency was measured using inpatient costs and quality was measured using the hospitalization rates with a primary diagnosis of type 2 diabetes. The number of regions as well as their sociodemographic characteristics are displayed in Supplementary Material 2. Given that the outcome hospitalization had a wider range than inpatient costs (Fig. 2), there were more regions that fell above or below the 20% threshold for quality than for efficiency (TopQual and BottomQual comprise 13 and 16 regions, while TopEff and BottomEff comprise 3 and 5 regions, see Supplementary Material 2).

Table 4 summarizes the average outcome value and the PopGroup-standardized outcome ratio for each parameter in the top and bottom regions. By definition, the TopEff regions have the lowest inpatient costs and the TopQual regions have the lowest hospitalization rate. The TopEff regions also exhibit hospitalization, amputation, and mortality rates below the national average, while the TopQual regions show lower rates of inpatient emergencies than the national averages. All outcomes in the BottomEff and BottomQual regions exceed the national average, indicating overall poorer performance.

**Table 4:** Average outcomes in the top and bottom regions in the year 2023

Region	Hospitalization		Inpatient emergency		Amputation		Inpatient costs		Inpatient treatment days		Mortality	
	%	O/E	%	O/E	%	O/E	Euros	O/E	Days	O/E	%	O/E
**All regions**	1.1%	1.0	0.7%	1.0	0.4%	1.0	3,324	1.0	4	1.0	5.1%	1.0
TopEff	0.9%	0.9	0.7%	1.1	0.3%	0.9	2,463	0.8	3	0.7	4.7%	1.0
BottomEff	1.3%	1.1	0.8%	1.0	0.5%	1.2	4,108	1.2	5	1.3	5.1%	1.0
TopQual	0.7%	0.7	0.5%	0.7	0.4%	0.9	3,061	0.9	4	0.9	5.3%	1.0
BottomQual	1.4%	1.3	0.9%	1.3	0.5%	1.1	3,368	1.0	4	1.0	5.2%	1.0

Note: O/E: observed to expected ratio. TopEff/BottomEff: Most and least efficient regions, measured using inpatient costs (20% above or below average). TopQual/BottomQual: Regions with highest and lowest quality, measured using hospitalization rates (20% above or below average). TopEff comprises 3 regions, BottomEff 5 regions, TopQual 13 regions and BottomQual 16 regions. All regions comprises all 96 regions.

Table 5 compares average utilization – using selected inpatient, ambulatory, and emergency care variables – during the year prior to the outcome measurement (2022) between the top- and bottom-performing regions, both averaged across all PopGroups and for the most populous PopGroup. The data show that total and inpatient costs, as well as inpatient treatment days, were already lowest in the TopEff regions in the previous year. Similarly, the BottomEff and BottomQual regions consistently perform worse than the top regions across nearly all inpatient utilization variables. The TopQual regions exhibit high outpatient costs across all PopGroups – for the most frequent PopGroup even the highest outpatient costs – while maintaining below-average inpatient and total costs.

**Table 5:** Average utilization in the top and bottom regions in the year 2022

	Region	N persons	Totel cost	Inpatient care					Ambulatory care					Emergency care		% in disease management program
				Cost		% with at least 1 hospital stay			Cost		Number of cases			Inpatient emergencies	Outpatient emergencies	
			Per person (Euros)	Per person (Euros)	% from total	With type 2 diabetes	All diagnoses	Treatment days	Per person (Euros)	% from total	Total	GP	Specialist	% with at least 1	% with at least 1	
All PopGroups	**All regions**	**745**,**741**	**5**,**511**	**2**,**558**	**46%**	**1.0%**	**25%**	**3.3**	**1**,**240**	**22%**	**15**	**5**	**8**	**15%**	**15%**	**59%**
	TopEff	6,931	4,946	2,033	41%	0.7%	21%	2.5	1,225	25%	14	5	7	13%	15%	57%
	BottomEff	27,856	5,888	3,051	52%	1.4%	29%	4.0	1,196	20%	14	5	7	17%	15%	52%
	TopQual	65,487	5,402	2,442	45%	0.9%	24%	3.0	1,255	23%	15	5	8	15%	15%	60%
	BottomQual	92,614	5,585	2,704	48%	1.2%	26%	3.5	1,184	21%	14	5	7	16%	15%	58%
Most frequent PopGroup	**All regions**	**101**,**814**	**2**,**838**	**584**	**21%**	**1.0%**	**11%**	**0.6**	**1**,**028**	**36%**	**14**	**6**	**7**	**5%**	**10%**	**80%**
	TopEff	984	2,483	403	16%	0.3%	9%	0.5	977	39%	13	5	6	4%	9%	78%
	BottomEff	3,941	2,950	852	29%	1.4%	15%	0.9	950	32%	14	6	6	6%	10%	72%
	TopQual	8,642	2,732	509	19%	0.7%	10%	0.5	1,046	38%	14	6	7	4%	10%	82%
	BottomQual	13,278	2,767	610	22%	1.2%	12%	0.7	969	35%	14	6	6	6%	10%	78%

Note: GP: General practitioner. Explanation of the columns: *Total cost* shows the average total expenditure per person in Euros. *Inpatient care*: *Cost* describes the inpatient cost per person in Euros and as a percentage of total cost. *% with at least 1 hospital stay* displays the percentage of the population with a hospital stay, first with type 2 diabetes and then with any principal diagnosis, as well as the average hospital treatment days. *Ambulatory care*: *Cost* describes the ambulatory cost per person in Euros and as a percentage of total cost. *Number of cases* shows the average number of total, GP and specialist cases per person. A “case” includes all services provided by a doctor’s office within a given quarter. Since the ambulatory care sector operates on a quarterly billing system, a case can represent either a single contact or multiple contacts within that quarter. *Emergency care* shows the percentage of the population with at least one inpatient or outpatient emergency. *% in disease management program* shows the percentage of the population enrolled in a disease management program for diabetes. TopEff/BottomEff: Most and least efficient regions, measured using inpatient costs (20% above or below average). TopQual/BottomQual: Regions with highest and lowest quality, measured using hospitalization rates (20% above or below average). TopEff comprises 3 regions, BottomEff 5 regions, TopQual 13 regions and BottomQual 16 regions. All regions comprises all 96 regions.

## Discussion

This paper quantified regional variation in diabetes-related efficiency and quality outcomes across Germany using the PopGrouper to adjust for morbidity; identified regions that performed above or below average based on PopGroup-adjusted performance; and explored patterns of care and resource utilization in top- and bottom-performing regions. The research demonstrates the utility of the PopGrouper as a tool for detailed risk-adjustment in regional health care comparisons, using diabetes care in Germany as a case example.

The findings reveal the greatest regional variation for amputations and emergency hospital admissions, followed by hospitalizations, for both age-sex-standardized and PopGroup-standardized ratios. These results are in line with prior research that found significant regional variations for these three outcomes, most of which reported their findings using age-sex standardization [4, 8, 11, 12, 15–19]. The observed variation was slightly lower with PopGroup standardization than with age-sex standardization, except in the case of mortality.

A key strength of this paper is the use of the PopGrouper for risk-adjustment. The PopGrouper is similar to existing international population classification systems (e.g., the ACG system [23] and the CRG system [24]), as it defines mutually exclusive groups that consider multimorbidity and exhibit a high degree of morbidity differentiation. However, the PopGrouper has been specifically developed within the German context and is publicly available [34]. Since PopGroups are mutually exclusive, they allow for a straightforward method of risk-adjustment through indirect standardization, similar to age-sex standardization, which is a well-established and widely used approach [32]. By applying this same approach with the PopGrouper, the methodology remains simple and accessible. This may be particularly beneficial for communicating with non-scientific audiences, as the method is less technical and more intuitive in its interpretation than complex regression analyses [22].

Beyond that, the PopGrouper’s high level of differentiation enables a very detailed measurement of morbidity. The PopGrouper integrates information from a wide range of health care services over an entire calendar year, including inpatient, outpatient, rehabilitation, prescription drugs, remedies, and medical aids. Furthermore, the PopGrouper takes into consideration the interactive impact of multimorbidity. This comprehensive approach to measuring morbidity may increase confidence that observed variations post-PopGroup standardization are not due to differences in population composition, and can serve as a foundation for future analyses of potential contributing factors (such as provider availability).

Given that all three events with the highest observed variations – amputations, emergency hospitalizations and hospitalizations – should be avoidable through timely and effective outpatient care, it is particularly important to investigate such regional differences. At the same time, it is important to note that the overall frequency of these events is low, meaning that even large variations often reflect small absolute differences. Still, such variations may reveal regions with higher quality of care and practices that support better management of diabetes. The identification of top-performing regions can provide valuable insights into best practices that other regions might be able to follow.

The paper reveals differences in utilization between the top- and bottom-performing regions. The top-performing regions had lower inpatient resource utilization, but higher outpatient resource utilization and participation in disease management programs in the year prior to the performance outcome measurement. This suggests that certain high-quality outpatient services may lead to better performance in the following year. These findings align with the definition of ACSCs and with prior research, which suggests that better outpatient management and continuity of care reduces the number of preventable complications and hospitalizations [21, 35].

Nonetheless, certain limitations need to be considered. First, this paper presents a simple and easily interpretable method to adjust for morbidity; however, it does not aim to fully explain the regional variation. This could for instance be achieved using a regression model that considers further factors such as the availability and access to providers.

Second, with the use of O/E ratios, regional performance is assessed relative to the national mean, using the national average as the benchmark for comparison. This eliminates the need for a normative “optimal” benchmark and allows a focus on the extent of regional differences. However, this approach limits any claims regarding “good” or “bad” performance and only allows for relative terms such as “better” or “worse”, relative to the national average.

Third, the use of data from a single sickness fund, which covers approximately 10% of the total German population, may limit the representativeness and generalizability of the results. The prevalence of diabetes among the BARMER population (8.4%) is slightly lower than the German national average of 10% in 2022 [36]. Hospitalization and amputation rates are also slightly lower in the BARMER population compared to the national averages – 1.1% vs. 1.7% for hospitalizations [37] and 0.4% vs. 0.6% for amputations [38], though the BARMER data refers to the year 2023, while the national data is only available for the year 2022. All-cause mortality appears to be higher among diabetes patients in the BARMER population, although the most recent national data dates to 2014, which limits comparability [39].

Fourth, in measuring performance, we used the PopGrouper based on health care utilization data from 2022 to adjust for morbidity and complexity. However, it is possible that certain comorbidities or complexities stem from poor diabetes management in previous years, which would imply that we over-adjust for poor regional performance. For this reason, the results should be interpreted as reflecting a region’s performance in 2023, based on the region’s morbidity structure in 2022.

Finally, the use of claims data comes with inherent limitations, such as coding inaccuracies, upcoding, or underdiagnosis, which may result in figures that do not accurately represent the true epidemiological data [40]. While the use of detailed selection and plausibility criteria – which are also used for the well-established risk-adjustment scheme among German sickness funds – helps mitigate the effects of upcoding, the possibility of underdiagnosis remains a limitation.

Overall, the primary aim of this research was to test the applicability of the PopGrouper and highlight its possible contribution when analyzing regional health care variation. Given the above-mentioned limitations, this paper does not aspire to offer specific recommendations for improving diabetes management. Instead, it demonstrates an additional analytical approach for health systems research, beyond the already existing morbidity measures. Performing the same analysis on a broader dataset from all German sickness funds, for instance in the newly launched national Health Data Lab [41], would give a better and more comprehensive overview of the true regional variations in diabetes care outcomes. In addition, future research can focus more on identifying best practice patterns that could contribute to improved results and lower regional variation.

In summary, the PopGrouper can make a significant contribution to controlling for morbidity-related care needs in regional comparative analyses. Regional variations in hospitalizations and related outcomes exist for diabetes patients in Germany, even after controlling for regional differences in morbidity.

## Supplementary Information

Below is the link to the electronic supplementary material.

Supplementary Material 1

Supplementary Material 2

## Data Availability

The data utilized in this current study are health insurance claims data provided by the German statutory health insurance fund BARMER. Due to legal and privacy restrictions, these data are not publicly available. Access to the data is subject to approval by the BARMER and may require additional agreements regarding data protection and confidentiality.
