# Supplementary material for: Regional variation in ambulatory care-sensitive hospitalizations for people with type 2 diabetes in Germany: insights from a claims data analysis using the PopGrouper
Source: Res Health Serv Reg. 2025 Dec 17;4:23. doi: 10.1007/s43999-025-00082-0 (PMC12711608; doi:10.1007/s43999-025-00082-0)
Supplement: Supplementary file 1 — Supplementary Material 1 [file 43999_2025_82_MOESM1_ESM.docx]

**Supplementary Material 1: Selection criteria for study population**

The study population was selected based on consolidated disease groups (CDGs) from the PopGrouper 1.0 [1]. CDGs build on diagnosis groups (DxGs) used in the German health insurance system allocate funds to health insurers based on morbidity [2]. The DxGs are based on ICD-10 codes from the International Statistical Classification of Diseases and Related Health Problems (10th Revision) and additional selection criteria as listed below:

|  |  |  |  |  |  |  |  |  |
| --- | --- | --- | --- | --- | --- | --- | --- | --- |
| **CDG** | **CDG description** | **DxG** | **DxG description** | **Selection criteria for DxG classifcation^1^** | | | | |
|  |  |  |  | **Age** | **ICD-10 codes** | **Inpatient diagnosis required^2^** | **Medication required^3^** | **Minimum treatment length** |
| Z0301 | Diabetes mellitus with diabetic ketoacidosis or coma | 96 | Other diabetes mellitus with diabetic ketoacidosis or coma | - | E11.0; E11.01, E11.1, E11.11, E12.0, E12.01, E12.1, E12.11, E13.0, E13.01, E13.1, E13.11, E14.0, E14.01, E14.1, E14.11 | special rule^1^ | special rule^1^ | special rule^1^ |
|  |  | 104 | Other diabetes mellitus with diabetic ketoacidosis or coma and long-term insulin medication | - | E11.0, E11.01, E11.1, E11.11, E12.0, E12.01, E12.1, E12.11, E13.0, E13.01, E13.1, E13.11, E14.0, E14.01, E14.1, E14.11, U69.73 | yes | no | no |
|  |  | 105 | Type 1 diabetes mellitus with diabetic ketoacidosis or coma | - | E10.0, E10.01, E10.1, E10.11 | yes | no | no |
| Z0302 | Diabetes mellitus with symptoms in at least one organ(-system) | 7 | Other diabetes mellitus with kidney involvement and long-term insulin medication | - | E11.2, E11.20, E11.21, E12.2, E12.20, E12.21, E13.2, E13.20, E13.21, E14.2, E14.20, E14.21 | special rule^1^ | special rule^1^ | special rule^1^ |
|  |  | 17 | Other diabetes mellitus with multiple complications and long-term insulin medication | - | E11.7, E11.72, E11.73, E11.74, E11.75, E12.7, E12.72, E12.73, E12.74, E12.75, E13.7, E13.72, E13.73, E13.74, E13.75, E14.7, E14.72, E14.73, E14.74, E14.75 | special rule^1^ | special rule^1^ | special rule^1^ |
|  |  | 54 | Other diabetes mellitus with symptoms of the nervous system and long-term insulin medication | - | E11.4, E11.40, E11.41, E12.4, E12.40, E12.41, E13.4, E13.40, E13.41, E14.4, E14.40, E14.41 | special rule^1^ | special rule^1^ | special rule^1^ |
|  |  | 75 | Other diabetes mellitus with symptoms of peripheral vessels and long-term insulin medication | - | E11.5, E11.50, E11.51, E12.5, E12.50, E12.51, E13.5, E13.50, E13.51, E14.5, E14.50, E14.51 | special rule^1^ | special rule^1^ | special rule^1^ |
|  |  | 76 | Other diabetes mellitus with other specified symptoms and long-term insulin medication | - | E11.6, E11.60, E11.61, E12.6, E12.60, E12.61, E13.6, E13.60, E13.61, E14.6, E14.60, E14.61 | special rule^1^ | special rule^1^ | special rule^1^ |
|  |  | 97 | Diabetic retinopathy | - | H36.0 | no | yes | >=183 days |
|  |  | 98 | Other diabetes mellitus with kidney involvement | - | E11.2, E11.20, E11.21, E12.2, E12.20, E12.21, E13.2, E13.20, E13.21, E14.2, E14.20, E14.21 | no | yes | >=183 days |
|  |  | 99 | Type 1 diabetes mellitus with kidney involvement | - | E10.2, E10.20, E10.21 | no | yes | >=183 days |
|  |  | 100 | Other diabetes mellitus with symptoms of the nervous system | - | E11.4, E11.40, E11.41, E12.4, E12.40, E12.41, E13.4, E13.40, E13.41, E14.4, E14.40, E14.41 | no | yes | >=183 days |
|  |  | 101 | Type 1 diabetes mellitus with symptoms of the nervous system | - | E10.4, E10.40, E10.41 | no | yes | >=183 days |
|  |  | 102 | Other diabetes mellitus with symptoms of peripheral vessels | - | E11.5, E11.50, E11.51, E12.5, E12.50, E12.51, E13.5, E13.50, E13.51, E14.5, E14.50, E14.51 | no | yes | >=183 days |
|  |  | 103 | Type 1 diabetes mellitus with symptoms of peripheral blood vessels | - | E10.5, E10.50, E10.51 | no | yes | >=183 days |
|  |  | 106 | Other diabetes mellitus with other specified symptoms | - | E11.6, E11.60, E11.61, E12.6, E12.60, E12.61, E13.6, E13.60, E13.61, E14.6, E14.60, E14.61, U69.74 | no | yes | >=183 days |
|  |  | 107 | Type 1 diabetes mellitus with other specified symptoms | - | E10.6, E10.60, E10.61 | no | yes | >=183 days |
|  |  | 108 | Other diabetes mellitus with ocular manifestations | - | E11.3, E11.30, E11.31, E12.3, E12.30, E12.31, E13.3, E13.30, E13.31, E14.3, E14.30, E14.31 | no | yes | >=183 days |
| **CDG** | **CDG title** | **DxG** | **DxG title** | **Selection criteria for DxG classifcation^1^** | | | | |
|  |  |  |  | **Age** | **ICD-10 codes** | **Inpatient diagnosis required^2^** | **Medication required^3^** | **Minimum treatment length** |
| Z0302 | Diabetes mellitus with symptoms in at least one organ(-system) | 109 | Type 1 diabetes mellitus with ocular manifestations | - | E10.3, E10.30, E10.31 | no | yes | >=183 days |
|  |  | 323 | Diabetic neuropathy | - | G59.0, G63.2 | no | no | no |
|  |  | 430 | Diabetic angiopathy | - | I79.2 | no | yes | >=183 days |
|  |  | 552 | Glomerular diseases in diabetes mellitus | - | N08.3 | no | yes | >=183 days |
|  |  | 800 | Other diabetes mellitus with multiple complications | - | E11.7, E11.72, E11.73, E11.74, E11.75, E12.7, E12.72, E12.73, E12.74, E12.75, E13.7, E13.72, E13.73, E13.74, E13.75, E14.7, E14.72, E14.73, E14.74, E14.75 | no | yes | >=183 days |
|  |  | 801 | Type 1 diabetes mellitus with multiple complications | - | E10.7, E10.72, E10.73, E10.74, E10.75 | no | yes | >=183 days |
|  |  | 802 | Other diabetes mellitus with unspecified complications | - | E11.8, E11.80, E11.81, E12.8, E12.80, E12.81, E13.8, E13.80, E13.81, E14.8, E14.80, E14.81 | no | yes | >=183 days |
|  |  | 803 | Type 1 diabetes mellitus with unspecified complications | - | E10.8, E10.80, E10.81 | no | yes | >=183 days |
|  |  | 1049 | Diabetic retinopathy with long-term insulin treatment | - | H36.0 | special rule^1^ | special rule^1^ | special rule^1^ |
|  |  | 1050 | Diabetic angiopathy in long-term insulin medication | - | I79.2 | special rule^1^ | special rule^1^ | special rule^1^ |
|  |  | 1051 | Diabetic neuropathy in long-term insulin medication | - | G59.0, G63.2 | special rule^1^ | special rule^1^ | special rule^1^ |
|  |  | 1052 | Glomerular diseases in diabetes mellitus with long-term insulin medication | - | N08.3 | special rule^1^ | special rule^1^ | special rule^1^ |
|  |  | 1066 | Other diabetes mellitus with ocular manifestations and long-term insulin medication | - | E11.3, E11.30, E11.31, E12.3, E12.30, E12.31, E13.3, E13.30, E13.31, E14.3, E14.30, E14.31 | special rule^1^ | special rule^1^ | special rule^1^ |
|  |  | 1067 | Other diabetes mellitus with unspecified complications and long-term insulin medication | - | E11.8, E11.80, E11.81, E12.8, E12.80, E12.81, E13.8, E13.80, E13.81, E14.8, E14.80, E14.81 | special rule^1^ | special rule^1^ | special rule^1^ |
| Z0303 | Diabetes mellitus without complications | 110 | Other diabetes mellitus without complications | - | E11.9, E11.90, E11.91, E12.9, E12.90, E12.91, E13.9, E13.90, E13.91, E14.9, E14.90, E14.91, O24.1, O24.2, O24.3 | no | no | no |
|  |  | 111 | Type 1 diabetes mellitus without complications | - | E10.9, E10.90, E10.91, O24.0 | no | yes | >=183 days |
|  |  | 1068 | Other diabetes mellitus without complications and long-term insulin medication | - | E11.9, E11.90, E11.91, E12.9, E12.90, E12.91, E13.9, E13.90, E13.91, E14.9, E14.90, E14.91, O24.1, O24.2, O24.3 | special rule^1^ | special rule^1^ | special rule^1^ |

Note: ^1^ based on [2]. ^2^ if “no”, then confirmed outpatient diagnosis in at least 2 quarters of the year is sufficient. ^3^ if “yes”, then at least 1 simultaneous medication prescription required. CDG: Consolidated disease Group

**References**

1. Tsatsaronis C, Klemt M, Kinder K, et al (2025) [Definition of consolidated disease groups for a population-based system to classify morbidity-related healthcare needs: PopGroup]. Gesundheitswesen 87:282–290. https://doi.org/10.1055/a-2541-9695

2. Bundesamt für Soziale Sicherung (2025) Risikostrukturausgleich: Festlegungen. https://www.bundesamtsozialesicherung.de/de/themen/risikostrukturausgleich/festlegungen/. Accessed 16 Jan 2025
