# Supplementary material for: Regional variation in ambulatory care-sensitive hospitalizations for people with type 2 diabetes in Germany: insights from a claims data analysis using the PopGrouper
Source: Res Health Serv Reg. 2025 Dec 17;4:23. doi: 10.1007/s43999-025-00082-0 (PMC12711608; doi:10.1007/s43999-025-00082-0)
Supplement: Supplementary file 2 — Supplementary Material 2 [file 43999_2025_82_MOESM2_ESM.docx]

**Supplementary Material 2: Sociodemographic characteristics of most and least efficient regions (TopEff, BottomEff) and the regions with the highest and lowest quality of care (TopQual, BottomQual)**

| **Rank** | **Region** | **Level of urbanization** | **Deprivation quintile** | **Population density** | **No of persons** | **Average age** | **Cases per 1,000** | **PopGroup severity** |
| --- | --- | --- | --- | --- | --- | --- | --- | --- |
|  |  |  |  |  |  |  |  |  |
| **TopEff** | | | | **164.3** | **2,310** | **71.1** | **77.8** | **0.06** |
| 1 | 346 | Urbanization | Lowest deprivation | 212.6 | 2,305 | 69.8 | 72.7 | 0.06 |
| 2 | 876 | Rural region | Second highest deprivation | 95.4 | 2,007 | 71.5 | 81.9 | 0.07 |
| 3 | 947 | Urbanization | Lowest deprivation | 185.0 | 2,619 | 71.9 | 78.9 | 0.06 |
| **BottomEff** | | | | **532.7** | **5,571** | **70.6** | **78.0** | **0.10** |
| 92 | 150 | Urban region | Highest deprivation | 1,117.2 | 9,203 | 70.0 | 85.8 | 0.11 |
| 93 | 731 | Urbanization | Medium deprivation | 172.6 | 4,645 | 70.3 | 72.9 | 0.09 |
| 94 | 319 | Urban region | Highest deprivation | 1,035.0 | 8,402 | 69.8 | 83.7 | 0.12 |
| 95 | 455 | Urbanization | Second lowest deprivation | 223.1 | 2,940 | 70.2 | 79.0 | 0.07 |
| 96 | 961 | Rural region | Lowest deprivation | 115.8 | 2,666 | 72.5 | 68.6 | 0.10 |
| **TopQual** | | | | **207.9** | **5,037** | **71.4** | **83.6** | **0.09** |
| 1 | 876 | Rural region | Second highest deprivation | 95.4 | 2,007 | 71.5 | 81.9 | 0.07 |
| 2 | 969 | Rural region | Lowest deprivation | 256.8 | 2,069 | 71.3 | 80.0 | 0.08 |
| 3 | 534 | Urbanization | Medium deprivation | 184.6 | 3,617 | 70.8 | 71.3 | 0.08 |
| 4 | 931 | Urbanization | Highest deprivation | 168.9 | 6,788 | 70.3 | 89.5 | 0.08 |
| 5 | 560 | Rural region | Lowest deprivation | 150.4 | 2,672 | 72.7 | 69.3 | 0.09 |
| 6 | 771 | Rural region | Second highest deprivation | 111.0 | 6,759 | 71.0 | 89.3 | 0.10 |
| 7 | 534 | Urban region | Second lowest deprivation | 433.1 | 10,757 | 70.8 | 85.7 | 0.06 |
| 8 | 996 | Urbanization | Second highest deprivation | 140.4 | 6,763 | 74.0 | 101.6 | 0.13 |
| 9 | 555 | Urban region | Lowest deprivation | 482.9 | 9,353 | 71.4 | 88.3 | 0.09 |
| 10 | 344 | Urbanization | Second highest deprivation | 213.9 | 4,749 | 71.0 | 84.2 | 0.09 |
| 11 | 902 | Urbanization | Medium deprivation | 159.8 | 2,683 | 71.1 | 77.2 | 0.08 |
| 12 | 926 | Urbanization | Lowest deprivation | 167.8 | 3,633 | 71.4 | 79.1 | 0.12 |
| 13 | 103 | Urbanization | Lowest deprivation | 138.6 | 3,637 | 70.9 | 90.0 | 0.12 |
| **BottomQual** | | | | **216.9** | **5,788.4** | **71.3** | **85.4** | **0.10** |
| 81 | 218 | Urbanization | Medium deprivation | 143.3 | 4,627 | 70.6 | 75.1 | 0.12 |
| 82 | 909 | Urbanization | Medium deprivation | 152.6 | 10,847 | 71.3 | 83.2 | 0.10 |
| 83 | 228 | Rural region | Second lowest deprivation | 140.8 | 3,536 | 71.1 | 95.0 | 0.10 |
| 84 | 576 | Urbanization | Second highest deprivation | 216.2 | 16,774 | 74.7 | 106.6 | 0.10 |
| 85 | 311 | Urbanization | Highest deprivation | 136.5 | 1,766 | 70.6 | 81.7 | 0.08 |
| 86 | 243 | Urbanization | Lowest deprivation | 196.1 | 3,362 | 71.3 | 84.6 | 0.08 |
| 87 | 178 | Rural region | Highest deprivation | 107.8 | 2,068 | 70.2 | 73.2 | 0.10 |
| 88 | 322 | Urbanization | Lowest deprivation | 178.6 | 1,526 | 71.9 | 84.9 | 0.14 |
| 89 | 150 | Urban region | Highest deprivation | 1,117.2 | 9,203 | 70.0 | 85.8 | 0.11 |
| 90 | 950 | Rural region | Second highest deprivation | 96.6 | 8,310 | 71.7 | 101.8 | 0.09 |
| 91 | 628 | Urbanization | Medium deprivation | 253.6 | 3,162 | 71.6 | 72.0 | 0.05 |
| 92 | 289 | Urbanization | Second lowest deprivation | 262.7 | 3,943 | 72.3 | 78.1 | 0.09 |
| 93 | 565 | Rural region | Second lowest deprivation | 110.6 | 5,852 | 71.2 | 83.5 | 0.11 |
| 94 | 634 | Rural region | Medium deprivation | 120.1 | 5,077 | 70.7 | 88.8 | 0.13 |
| 95 | 404 | Rural region | Highest deprivation | 64.9 | 7,916 | 71.6 | 99.6 | 0.10 |
| 96 | 731 | Urbanization | Medium deprivation | 172.6 | 4,645 | 70.3 | 72.9 | 0.09 |

Note: Top and bottom regions measured as regions 20 % above or below average. Deprivation measured using the German Index of Socioeconomic Deprivation (GISD) [1]. Population density per km^2^. Cases per 1,000 are age-sex-standardized.

**References**

1. Michalski N, Reis M, Tetzlaff F, et al (2022) German Index of Socioeconomic Deprivation (GISD): Revision, Aktualisierung und Anwendungsbeispiele. https://doi.org/10.25646/10640
